# Shenqi Fuzheng injection hinders non-small cell lung cancer cell growth by regulating the Bax/Bcl-2 signaling pathway

**DOI:** 10.1007/s12672-024-01029-6

**Published:** 2024-05-29

**Authors:** Siqi Li, Tianyu Ma, Gege Li, Xu Cheng, Tao Wen, Yuxuan Wang, Hongtao Zhang, Zhidong Liu

**Affiliations:** 1 No. 2 Department of Thoracic Surgery, Beijing Chest Hospital/Beijing Tuberculosis and Thoracic Tumor Research Institute, Capital Medical University, Beijing, China; 2 Department of Thoracic Surgery, Beijing Anzhen Hospital, Capital Medical University, Beijing, China; 3 Cancer Research Center, Beijing Tuberculosis and Thoracic Tumor Research Institute/Beijing Chest Hospital, Capital Medical University, Beijing, China

**Keywords:** Shenqi Fuzheng injection, Non-small cell lung cancer, Antitumor, Apoptosis

## Abstract

**Introduction:**

Lung cancer (LC) is the most common solid tumor and is currently considered the primary cause of cancer-related deaths worldwide. In clinical efficacy studies, it was not difficult to find that the combination of SFI and chemotherapy could improve the general condition of patients, reduce the side effects of chemotherapy drugs, and have a cooperative antitumor effect in NSCLC patients. However, whether SFI can be used as a novel antitumor drug is still unknown.

**Methods:**

First, meta-analysis aimed to explore the efficacy of SFI in NSCLC patients, and SFI was identified by ultra-performance liquid chromatography‒mass spectrometry (UPLC‒MS). Cell proliferation, migration, and invasion were explored by Cell Counting Kit-8 (CCK-8), scratch healing, and Transwell assays, respectively. Cell cycle and apoptosis assays were performed by flow cytometry. Transcriptome sequencing analysis was performed in four NSCLC cell lines. Differential expression analysis was used to identify potential targets. Apoptosis-related protein levels were detected by Western blotting assays. The effects of SFI in NSCLC were further investigated by mouse xenografts.

**Results:**

SFI could markedly improve the chemotherapy efficacy of NSCLC patients. The main active ingredients include flavonoids and terpenoids, which can effectively exert antitumor effects. SFI could not only inhibit tumor cell proliferation and cell migration/invasion but also regulate the cell cycle and promote tumor cell apoptosis. In NSCLC, SFI could enhance the transcription level of the CHOP gene, thereby upregulating the expression of the proapoptotic proteins Bax and caspase 3, and inhibiting the expression of the antiapoptotic protein Bcl-2. SFI hindered the growth of mouse NSCLC xenografts in vivo.

**Conclusions:**

SFI hindered tumor progression and might promote apoptosis by increasing the expression of Bax, caspase 3 and decreasing the level of Bcl-2 in NSCLC.

**Supplementary Information:**

The online version contains supplementary material available at 10.1007/s12672-024-01029-6.

## Introduction

Lung cancer (LC) is the most common solid tumor and is currently considered the primary cause of cancer-related deaths worldwide. In 2022, there will be an average of 236,740 new cases and 130,180 deaths [1] non-small cell lung cancer (NSCLC) accounts for 80–85% in LC patients, and treatment options are diverse, including surgery, radiotherapy, chemotherapy, targeted therapy and immunotherapy, etc. Although new treatments such as targeted therapy and immune checkpoint inhibitors have significantly improved the treatment effect of NSCLC patients, the current status of curative effect in lung cancer patients is still unsatisfactory, and the overall 5-year survival rate is approximately 19.3% [2, 3]. Therefore, it is still extremely important to discover new therapeutic strategies or antitumor drugs.

In China, Shenqi Fuzheng Injection (SFI), a decoction mainly mixed with Astragalus and Codonopsis extracts, was approved by the State Food and Drug Administration of China in 1999 as an antitumor treatment [4, 5]. Radix Astragali, the dried root of Astragalus membranaceus (Fisch.) Bge. var. mongholicus (Bge.) Hsiao, has been used as a treatment for overall weakness, persistent disease and spleen deficiency including anorexia, asthenia, and diarrhea [6]. Furthermore, studies have shown that Radix Astragali has immunomodulatory, antioxidant, anti-inflammatory, and antitumor effects [7, 8].

Of note, many clinical trials of SFI in combination with platinum have been conducted with NSCLC, breast cancer and other cancerous patients [9, 10]. In clinical efficacy studies, it was not difficult to find that the combination of SFI and chemotherapy could improve the general condition of patients, reduce the side effects of chemotherapy drugs, and have a cooperative antitumor effect in NSCLC patients [11, 12]. However, whether SFI can be used as a novel antitumor drug is still unknown. The potential targets and antitumor mechanisms of SFI are still catching the eyes of many investigators.

To better utilize SFI to treat NSCLC, we further studied the effect and molecular mechanism of SFI on the progression and apoptosis of NSCLC cells in vivo and in vitro. We investigated the effect of SFI on the proliferation, migration, invasion, cycle and apoptosis of NSCLC cell lines. To identify the potential targets of SFI, SFI-treated tumor cells were subjected to transcriptomic sequencing, and based on differential expression analysis, it was found that CHOP in NSCLC may be a potential target gene of SFI. The enrichment analysis of the KEGG pathway found that the CHOP gene was significantly correlated with the apoptosis pathway. We further explored whether SFI could suppress tumor growth by regulating the levels of apoptosis-related proteins. In addition, the effect of SFI in NSCLC tumorigenesis was further explored in vivo. In our study, we found that SFI could impede the progression of NSCLC cell lines in vivo and in vitro, and might promote the apoptosis of NSCLC cells by regulating the expression of the potential target CHOP.

## Materials and methods

### Reagents

Acetonitrile (HPLC grade) was purchased from Fisher Scientific (Fairlawn). Apoptosis kits and cell cycle kits were purchased from Abcam (Shanghai, China). The RNA Prep Pure Cell/Bacteria Kit (DP430) was purchased from TIANGEN (Beijing, China). GAPDH Rabbit mAb (A19056), Bcl-2 Rabbit mAb (A19693), Bax Rabbit mAb (A19684), Caspase-3 Rabbit mAb (A19654) were purchased from ABclonal (Wuhan, China). All reagents for Western Blotting were purchased from Beyotime Biotechnology (Shanghai, China), and SFI was provided by Livzon Pharmaceuticals Ltd. (Guangdong, China).

### Cells and animals

The lung adenocarcinoma (LUAD) cell lines A549 and NCI-H2009, and the lung squamous carcinoma (LUSC) cell lines NCI-H226 and SK-MES-1 were purchased from the Institute of Chinese Academy of Medical Sciences, Cell Center (Beijing, China). A549, NCI-H2009 and NCI-H226 cells were cultured in RPMI-1640 medium (03.4007C, EallBio, Beijing) supplemented with 10% FBS and 1% P/S. SK-MES-1 cells were nourished in MEM medium (03.10001C, EallBio, Beijing) plus 10% FBS and 1% P/S. All cell lines were routinely maintained in 37 °C, 5% CO_2_ thermotank.

Male BALB/c nude mice (4–6 weeks old, 16–18 g) were purchased from the Laboratory Animal Centre of Beijing Chest Hospital, Capital Medical University, and were kept under pathogen-free conditions. All animal experimental procedures were carried out following the Beijing Municipal Experimental Animal Management Committee and the Capital Medical University Animal Experimentation Ethics Guidelines.

### META analysis

PubMed, Embase, Web of Science, Cochrane Library, CNKI and SINOMED databases were listed for all related articles published until 30 May 2022. This study aimed to analyze all randomized clinical trials that compared the efficacy of SFI in combination with chemotherapy versus chemotherapy alone in the treatment of NSCLC. The subject and free searching terms were Shenqi Fuzheng Injection, Non-small cell lung cancer, chemotherapy. Two researchers worked independently on study selection, data extraction and synthesis. The quality of the studies was assessed by RoB2. The leading study endpoint is the objective response rate (ORR). RevMan 5.4 software was used to analyze the results.

### Separation and analysis of active compounds in Shenqi Fuzheng injection

Chemdraw 2020 software was used for compound structure drawing. The samples were separated with Waters BEH C18 column (2.5 μm, 2.1 mm × 150 mm) using Thermo Scientific (U3000) system consisting of SCIEX 5600 + mass spectrometer. The eluents were (A) 0.1% HCOOH-H2O and (B) acetonitrile. The gradient elution of the mobile phase was 95% (A) in 0–2 min, 95–5% (A) in 2–10 min, 5% (A) in 10–13 min, and 5–95% in 13–15 min at a flow rate of 0.3 mL/min. For UPLC‒MS analysis, the UPLC conditions were the same as above. Ultra-high and pure helium (He, 99.999%) was used as the collision gas, and high purity nitrogen (N2, 99.999%) was used as the nebulizing gas. Positive and negative ion modes ESI–MS and MS/MS were used for the detection. TOF MS parameters: source temperature: 500 °C (positive ion) and 450 °C (negative ion), scan range, m/z = 50–1200, scan accumulation time 0.2 s, product ion scan accumulation time 0.01 s; MS/MS parameters: high sensitivity mode with information-dependent acquisition (IDA), declustering potential (DP): ± 60 V, collision energy: 35 ± 15 eV.

### Cell viability assay

Cell proliferation was assessed by the cell counting kit 8 (CCK-8) assay. Cells were grown in 96-well culture plates at 8 × 10^**3**^ cells/well and treated with SFI at different drug concentrations (0, 0.04, 0.08 g/ml) for 24, 48 and 72 h. After treatment, the cells were incubated for 1 h at 37 °C with CCK-8 reagent (03.17002DA, EallBio). The absorbance of 96-well culture plates was tested at 450 nm by enzyme-linked immunoassay (Thermo, USA). All experiments were repeated at least three times.

### Cell cycle assay

A549, NCI-H226, NCI-H2009, and SK-MES-1 cells were treated with SFI for 72 h. The cells were collected, washed in PBS and fixed at 4 °C in 75% ethanol overnight. Then, 500 μl of PI/RNase (550825, BD Pharmingen) staining solution was added, incubated at 25 °C for 15 min in the dark and analyzed by flow cytometry (BD, USA).

### Apoptosis assay

Cells were treated for 72 h with SFI as the experimental group or cell culture medium as the control group. Cell lines were collected in 500 μl of binding buffer, 7-AAD and Annexin V (556547, BD Pharmingen) were added and incubated at 25 °C for 15 min in the dark, and apoptosis was evaluated using flow cytometry (BD, USA).

### Scratch test

Cells were inoculated into 6-well plates at 1 × 10^5^ cells/well, and a scratch wound was created using a 20 μl pipette tip when cell confluence reached approximately 80%. PBS was rinsed to remove floating cells and debris, and treated with SFI and serum-free medium. Wound healing was recorded at 24 and 48 h after the formation of the scratch and repeated three times. Relative area = (scratch area of 0 h/24 h/48 h)/scratch area of 0 h × 100%.

### Invasion experiments

Matrigel Transwell analysis was performed to assess the invasive capacity of NSCLC cells. Then, 100 μl of Matrigel solution (122799, PerkinElmer) was added and incubated for 1 h. A total of 1 × 10^4^ cells were seeded into the upper chamber of a 24-well transwell plate. The upper chamber was added with serum-free medium, and the lower chamber with SFI and serum-free medium. Then, cells that invaded to the bottom side of the upper chamber were fixed and stained with crystalline violet. Finally, the cells were imaged under an inverted microscope.

### Transcriptome sequencing

RNA was extracted from the cells using the RNA Prep Pure Cell/Bacteria Kit (DP430, Tiangen, Beijing, China). Then, the quality of the extracted RNA was measured using a UV spectrophotometer. To generate mRNA-seq libraries, sequencing was performed using the DNBSEQ platform (UW Genetics, Shenzhen) according to the manufacturer's instructions.

### Protein extraction and western blotting

To assess protein expression in cells, treated cells were extracted with RIPA lysate (P0013B, Biotronik Biologicals) spiked with cocktail (P1048, Biotronik Biologicals). The cells were treated with SFI for 72 h. Equal amounts of protein samples (30–40 μg) were electrophoresed on 4–15% SDS—polyacrylamide gels (C621104, Biotech), transferred to PVDF membranes and milk closed. Subsequently, incubation with primary and secondary antibodies was performed. Immunoblot signals were detected by ECL, and the expressed proteins were quantified by ImageJ software.

### Establishment of a nude mouse xenograft model

In general, BALB/c nude mice were injected hypodermically with 5 × 10^6^ A549 cells/mouse. One week later, the mice were randomly allocated into 2 groups (n = 5) in which the tumor size reached approximately 100 mm^3^. The experimental group was injected intraperitoneally with 5 mg/kg every day for a fortnight. The control group was treated with PBS. Tumor volume and body weight were measured every other day, and tumor volume followed the standards: tumor volume (mm^**3**^) = 0.5 × (tumor width) ^2^ × tumor length. The mice were mercy killed following the Animal Experimentation Ethics Guidelines, and then the tumors were removed, photographed and weighed.

### Statistical analysis

All results are expressed as the mean ± standard deviation (S.D.). Statistical analysis was performed using Student’s t test (for two groups of data) or one-way ANOVA. Additionally, analyses were performed using the Wilcoxon rank sum test with Bonferroni correction, if needed. *p < 0.05 is a statistically significant difference; **p < 0.01, ***p < 0.001; NSD, no significant difference. All statistical analyses were performed using Review Manager 5.4 (Cochrane, UK), Stata (Computer Resource Center, USA), GraphPad Prism 9.0 (GraphPad, USA) and SPSS 20.0 software (SPSS, USA). Chemdraw 2020 software was used for compound structure drawing.

## Results

### SFI helps to improve the treatment effect of NSCLC

**Fig. 1:**
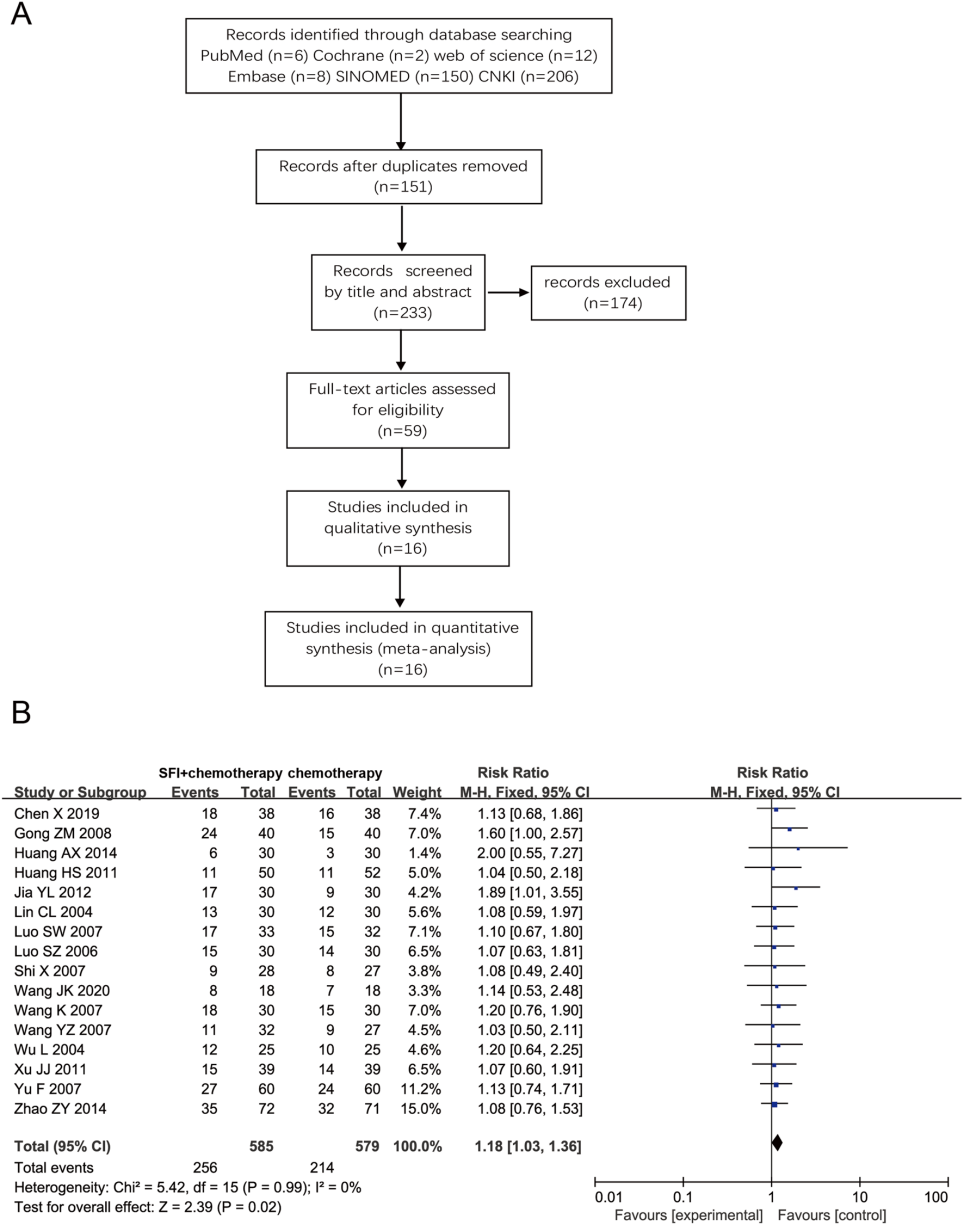
SFI improved the treatment outcome in NSCLC. **A** Flow Diagram of Searching for Eligible Studies. **B** Forest plot of objective tumor response. *SFI* Shenqi Fuzheng Injection, *NSCLC* non-small cell lung cancer

To study the efficacy of SFI in NSCLC patients, we screened relevant randomized controlled trials by searching PubMed, Cochrane, Web of Science, Embase, SINOMED, CNKI and other databases and conducted a meta-analysis. In the literature screening process (Fig. 1A), the search terms included the subject and free words "non-small cell lung cancer", "Shenqi Fuzheng Injection" and "chemotherapy". After screening, a total of 16 eligible randomized controlled trials were included, covering 1,164 patients (supplementary Table 1). Of them, fourteen studies were of low risk, and two were of some concern, according to the ROB2 Risk of Bias tool (supplementary Fig. 1A). Funnel plots (supplementary Fig. 1B) and Egger's linear regression analysis (P = 0.60) indicated that there was no significant publication bias between studies. Meta-analysis results showed that compared with the chemotherapy control group, the ORR of SFI combined with chemotherapy was significantly higher (RR = 1.18, 95% CI 1.03–1.36, P = 0.02, Fig. 1B). The above findings suggest that SFI can effectively improve the chemotherapy efficacy of NSCLC patients and has a synergistic antitumor effect.

### Identification of bioactive compounds in SFI

**Fig. 2:**
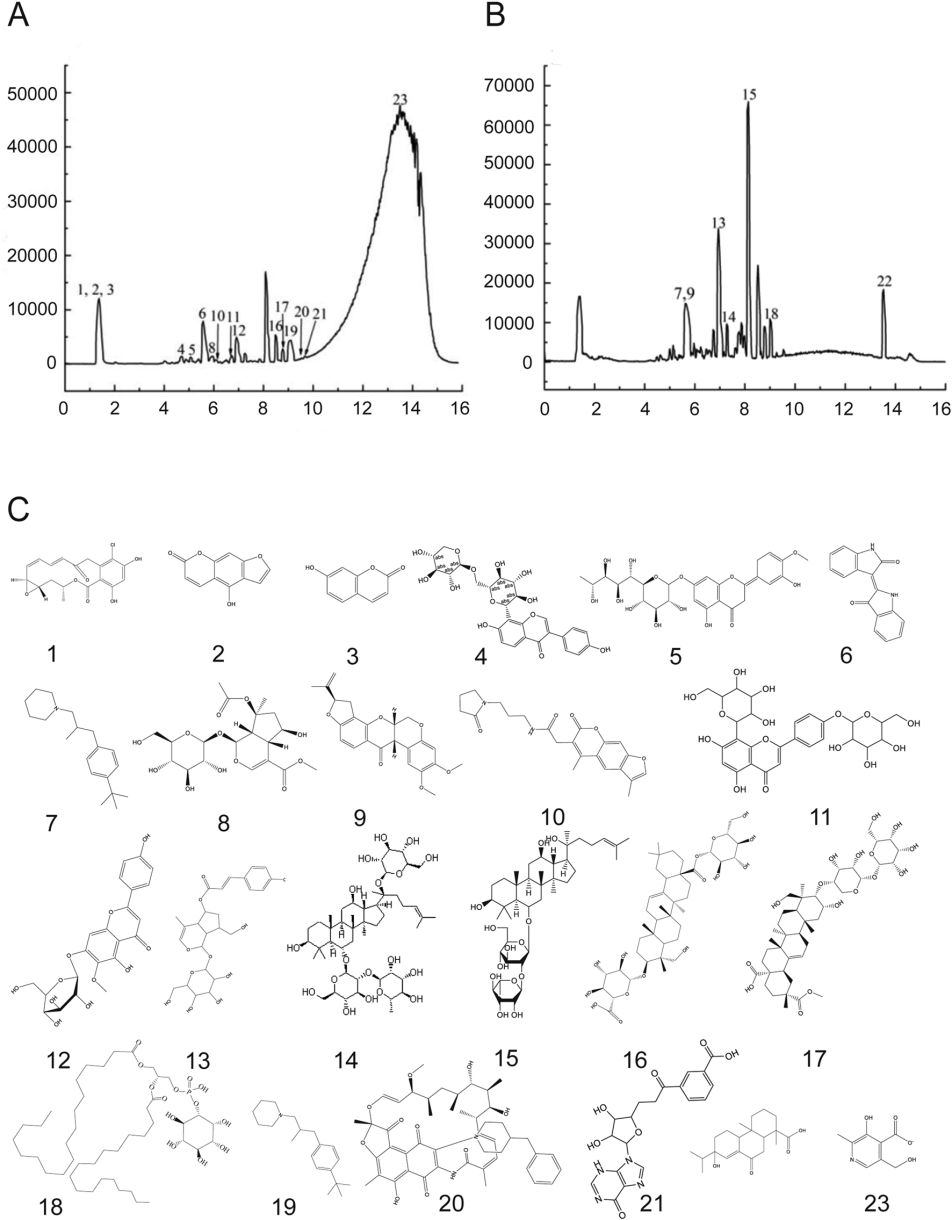
The identification of bioactive compounds in SFI. **A** UPLC fingerprint chromatograms of SFI. **B** Compounds of SFI. **C** Scheme of the compounds structrue. *SFI* Shenqi Fuzheng Injection, *UPLC* ultra-performance liquid chromatography

For the identification of biologically active components present in SFI, UPLC‒MS/MS was a prominent approach to solving the chemical structure in extract solution from traditional Chinese medicine because mass spectrometry provides abundant structural information, which was beneficial to the identification of unknown compounds. Essential Science Indicators (ESI) was used to examine bioactive compounds, and the negative mode was more sensitive than the positive mode (Fig. 2A). Through a full search of MassBank, Respect, and GNPS, after comparing the extracted peak information with the database, 23 bioactive compounds were analyzed and identified (Fig. 2B, C, Supplementary Table 2). The activities of the 23 compounds involved many aspects, among which compounds 2, 3, 4, 5, 6, 7, 8, 10, 11, 12, 13, 14, 15, 16, 17, and 22 were related to antitumor activity, so the next experiments were about the antitumor activity and modulation mechanism of SFI.

### SFI inhibits the growth of NSCLC cells and promotes apoptosis

**Fig. 3:**
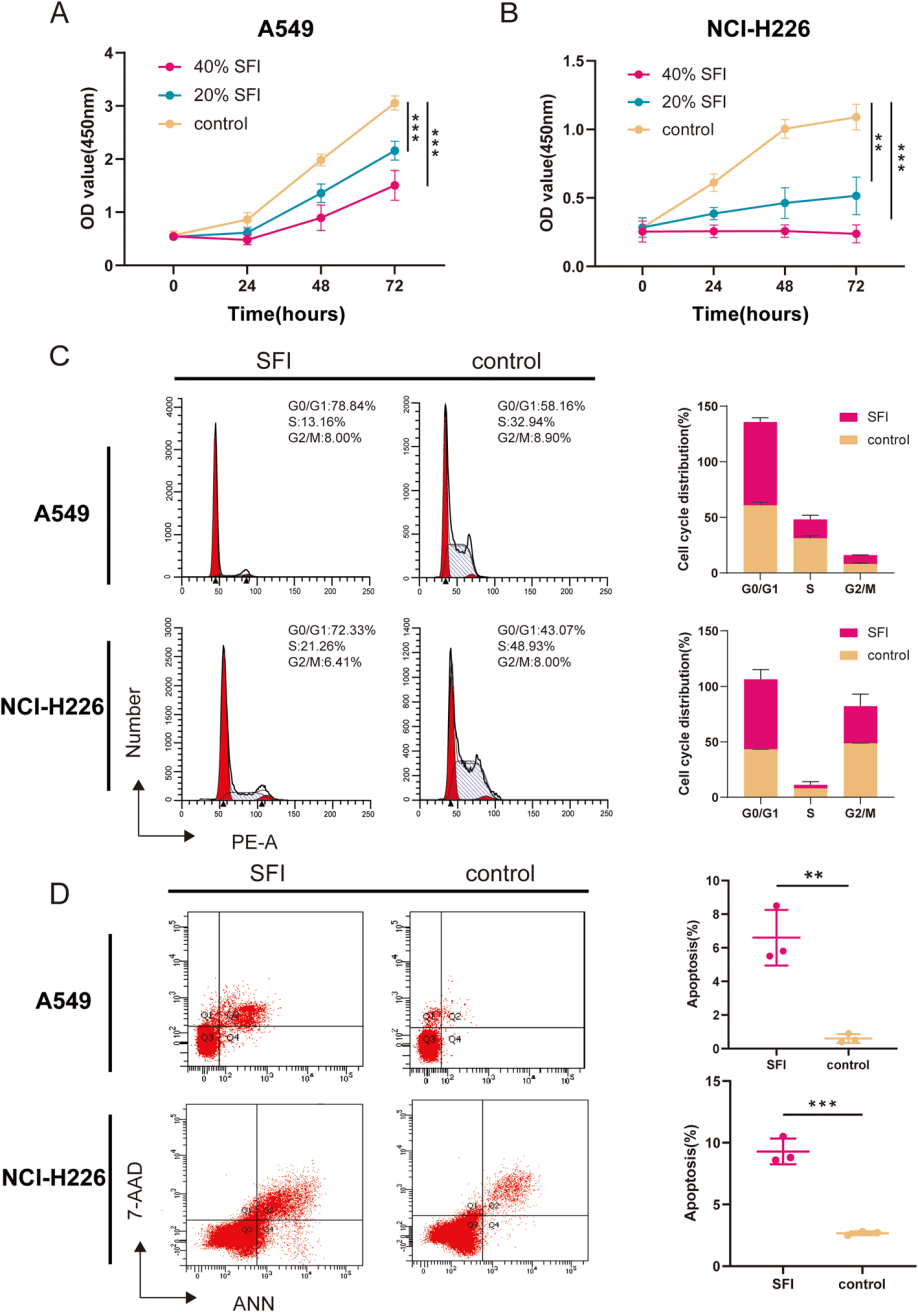
SFI inhibited growth and promoted cell apoptosis in NSCLC. A549 (**A**) and NCI-H226 (**B**) cells viability were measured by cell counting kit 8 assay after treatment with SFI at different concentrations for 24, 48, and 72 h. **C** Cells were cultured in medium with SFI for 72 h, fixed with cold ethanol overnight, and incubated with PI/RNase staining buffer for 15 min. Cell cycle distribution was analyzed by flow cytometry. **D** The apoptosis of SFI-treated A549 and NCI-H226 cells was analyzed by flow cytometry. *p < 0.05, **p < 0.01, ***p < 0.001. *SFI* Shenqi Fuzheng Injection, *NSCLC* non-small cell lung cancer

To further investigate the inhibitory effect of SFI in tumor cells, we focused on the apoptosis, cell cycle, cell proliferation, migration and invasion assays of NSCLC cell lines. We first treated NSCLC cells with different doses of SFI (0, 0.04, 0.08 g/ml), and analyzed the effect of SFI on the proliferation of NSCLC cells with CCK-8. The results showed that compared with the control group, A549 and NCI-H226 cells viability in the SFI group decreased significantly (P < 0.001, Fig. 3A, B). Moreover, SFI also significantly inhibited the proliferation of NCI-H2009 and SK-MES-1 cells (P < 0.001, supplementary Fig. 2A, B). SFI inhibited the proliferation of NSCLC cells in time and dose dependent manner.

To further explore the biological function of SFI on tumor cell lines, we investigated the effect of SFI on apoptosis and the cell cycle of NSCLC cells. The results of the cell cycle assay showed that SFI could induce A549 (P < 0.01) and NCI-H226 cells (P < 0.05, Fig. 3C) to arrest at G0/G1 phase, thereby regulating the cell cycle. These results indicated that SFI could block A549 and NCI-H226 cells in the G0/G1 phase, which may be one of the reasons for its inhibition of cell proliferation. In addition, Annexin V-FITC/7-AAD staining showed that SFI could significantly promote the apoptosis of A549 and NCI-H226 cells. When the concentration was 0.08 g/ml, the apoptosis rate of A549 cells was approximately 7% (P < 0.01) and that of NCI-H226 cells was approximately 10% (P < 0.001, Fig. 3D). Similarly, SFI significantly promoted apoptosis of NCI-H2009 cells (P < 0.01), and SFI mildly promoted apoptosis of SK-MES-1 cells (P > 0.05, supplementary Fig. 2C). The above results suggest that SFI can inhibit the proliferation of NSCLC cells, regulate the cell cycle and induce apoptosis.

### SFI inhibits NSCLC cell migration and invasion

**Fig. 4:**
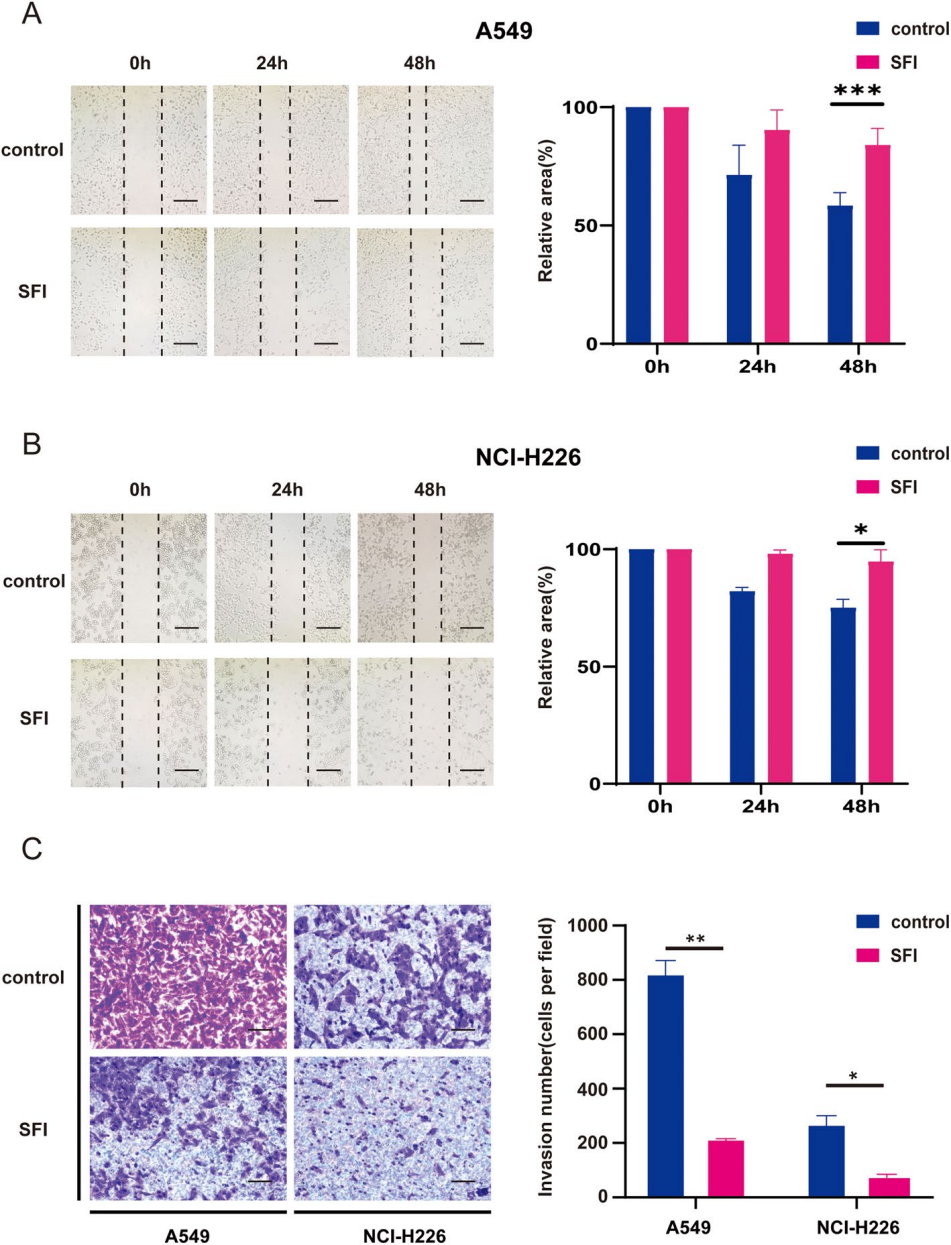
SFI inhibited migration and invasion in NSCLC. A549 (**A**) and NCI-H226 (**B**) cells were treated with SFI for the migration assay. **C** A549 and NCI-H226 cells invasion were measured using the Transwell system as described in Materials and Methods. *p < 0.05, **p < 0.01, ***p < 0.001. *SFI* Shenqi Fuzheng Injection, *NSCLC* non-small cell lung cancer

At the same time, we explored the effect of SFI on cell migration and invasion through scratch and Transwell assays. The wound healing assay results showed that SFI significantly reduced the migration distance of A549 and NCI-H226 cells (Fig. 4A, B). In A549 cells, the inhibition rates of the 0.08 g/ml group at 24 h and 48 h were 28.7 ± 10.3(%) and 41.7 ± 4.5(%) (P < 0.001, Fig. 4A). Similarly, the inhibition rates of 0.08 g/ml for 24 h and 48 h were 18.0 ± 1.4 (%) and 25.0 ± 2.9 (%) in NCI-H226 cells, respectively (P < 0.05, Fig. 4B). In addition, SFI also significantly inhibited the migration of NCI-H2009 (P < 0.05) and SK-MES-1 cells (P < 0.01, supplementary Fig. 3A, B).

Transwell results also showed that SFI had an inhibitory effect on the distant invasion of A549 and NCI-H226 cells (Fig. 4C). After 24 h of A549 cells, the number of cells that successfully passed through the Matrigel was 824.0 ± 56.7 cells in the control group and 216.0 ± 8.5 cells in the SFI group (P < 0.01). After NCI-H226 cells acted for 24 h, the number of cells that successfully passed through the filter was 270.5 ± 38.9 cells in the control group and 79.0 ± 12.7 cells in the SFI group (P < 0.05). Likewise, SFI significantly inhibited the migration and invasion of NCI-H2009 (P < 0.01) and SK-MES-1 cells (P < 0.05, supplementary Fig. 3C). These results suggest that SFI has inhibitory effects on the migration and invasion of NSCLC cells.

### SFI promotes tumor cell apoptosis by regulating the expression of CHOP

**Fig. 5:**
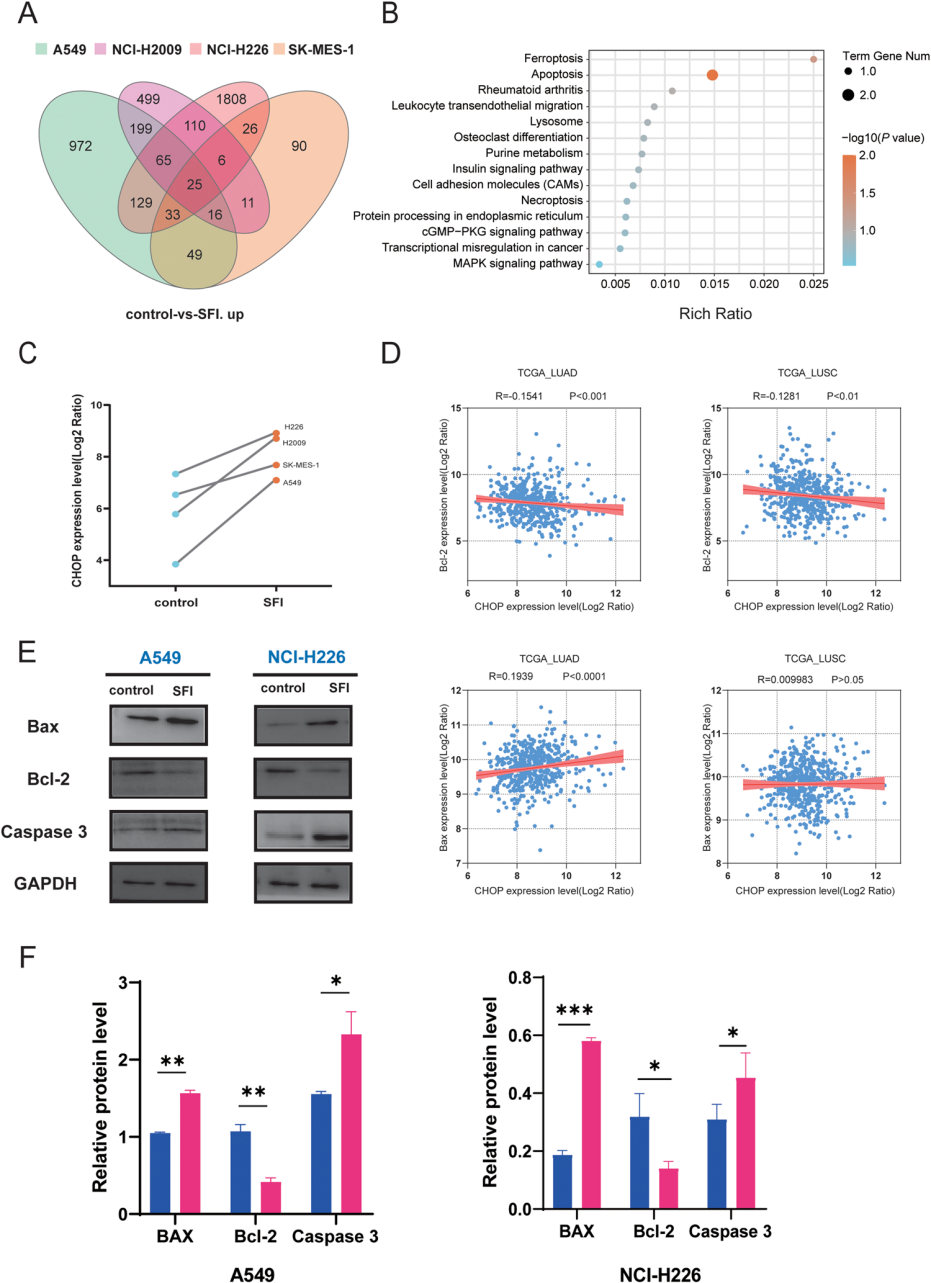
SFI promoted tumor cell apoptosis by regulating the expression of CHOP. **A** Venn diagram of a number of up-regulated genes at the mRNA level in SFI-treated NSCLC cells. **B** The KEGG pathways are enriched by upregulated genes in the SFI-treated cells compared with the control cells. **C** CHOP expression level in the SFI or control group. **D** The relationship between Bcl-2/Bax and CHOP in TCGA-LUAD and TCGA-LUSC datasets. **E**, **F** The expression levels of Bax, Bcl-2, and Caspase 3 were measured by Western blotting in A549 and NCI-H226 cells treated with SFI. *p < 0.05, **p < 0.01, ***p < 0.001. *SFI* Shenqi Fuzheng Injection, *NSCLC* non-small cell lung cancer, *KEGG* Kyoto Encyclopedia of Genes and Genomes, *TCGA* The Cancer Genome Atlas, *LUAD* lung adenocarcinoma, *LUSC* lung squamous carcinoma

In order to determine the possible functional targets of SFI, mRNA-seq was used to compare the transcriptional levels of A549, NCI-H226, NCI-H2009 and SK-MES-1 cells before and after SFI treatment. The mRNA-seq results showed that the expression levels of the same 25 genes were significantly upregulated in the different types of cells treated with SFI compared with the control group (Fig. 5A). Kyoto Encyclopedia of Genes and Genomes (KEGG) enrichment analysis showed that upregulated genes (CHOP, CTSK and SAT1, etc.) were associated with apoptosis and ferroptosis pathways. Based on the KEGG enrichment results, we suspected that SFI was most likely involved in the regulation of apoptosis signaling pathways (Fig. 5B). We analyzed and compared the expression of CHOP, and the level of CHOP was significantly raised after SFI treatment (P < 0.05, Fig. 5C). Combined with the Cancer Genome Atlas (TCGA) database analysis of LUAD and LUSC, we found that CHOP is closely related to the anti-cell apoptosis gene Bcl-2 was negatively correlated and positively correlated with the pro-apoptosis gene Bax (P < 0.05, Fig. 5D). Finally, Western blotting results showed that SFI could downregulate the expression level of the anti-apoptotic protein Bcl-2 and upregulate the expression of the pro-apoptotic proteins Bax and caspase 3 in A549 and NCI-H226 cells (P < 0.05, Fig. 5E, F). In summary, SFI could promote apoptotic progression in A549 and NCI-H226 cells.

### SFI inhibits the growth of xenograft tumors in mice

**Fig. 6:**
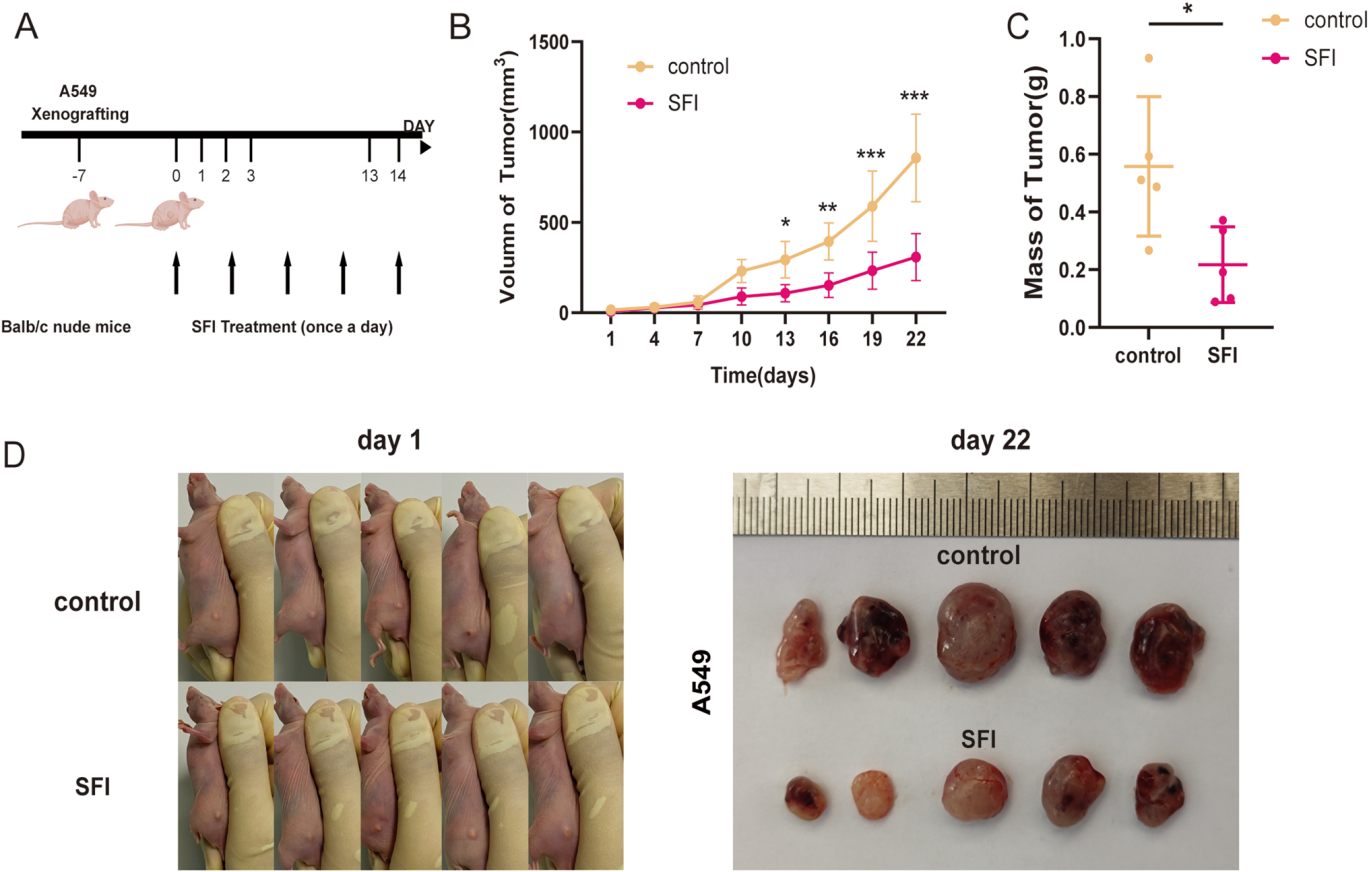
SFI inhibited the growth of NSCLC xenografts derived from A549 cells. A549 cells were subcutaneously injected into the flanks of mice to establish xenograft models. **A** When the volume of xenograft tumors reached approximately 100 mm^**3**^, mice received treatment with SFI (5 mg/kg) via intraperitoneal injection per day. **B** Tumor volume was measured every 3 days after SFI treatment. Tumor volume (**B**, **D**) and weight (**C**) were determined 14 days after SFI treatment. *p < 0.05, **p < 0.01, ***p < 0.001. *SFI* Shenqi Fuzheng Injection, *NSCLC* non-small cell lung cancer

To further study the effect of SFI in vivo, tumor xenograft experiments were carried out in BALB/c nude mice. Tumor volume was measured 14 days after dosing. As shown in Fig. 6A, the tumor volume in the SFI group was significantly smaller than that in the control group (P < 0.001, Fig. 6B, D). At the end of the experiment, the total weight of the transplanted tumors in the SFI group was significantly lighter than that in the control group (P < 0.01, Fig. 6C). These results confirm that SFI could obviously restrain the growth of tumor xenografts.

## Discussion

Lung cancer remains the leading cause of cancer death, with a 5-year survival rate of only 10%-20% in most countries, which has been a major challenge for NSCLC [13]. To treat NSCLC more comprehensively, we hope to discover natural antitumor components from traditional Chinese medicine to enrich tumor treatment methods. Natural products have a durable and plenty history in using tumor treatment and have been increasingly used as complementary and alternative therapies [14]. The natural products of anticancer drugs have been used in clinical treatment [15].

SFI is a Chinese herbal formula consisting of Codonopsis pilosula and Astragalus, which have been used as herbal medicines in China and other Asian countries for years [9]. In China, Astragalus is commonly used as an immunomodulatory agent to treat immunodeficiency symptom and to decrease the side effects of chemotherapy drugs [16, 17]. Studies have shown that Codonopsis is often used for antitumor, immune regulation, etc. [18]. SFI is made of Astragalus and Codonopsis, which indicates that it might have both the pharmacological activities of these herbs and has a more complete effect in cancer therapy. In addition, a recent study showed that adjuvant therapy for SFI could improve the resistance of NSCLC to EGFR-TKIs via the MAPK/SREBP1 pathway [19]. It has also been reported that SFI can inhibit A549/DDP cells and induce apoptosis [6]. In this study, we found that SFI inhibited the growth of LUAD and LUSC cell lines in vitro and in vivo, and indicated that SFI might provide insight into the development of new anti-NSCLC lead compounds.

At present, many clinical meta-analyses on SFI have found that SFI intervention could improve the tumor efficacy [20, 21], enhance the condition of patients, and decrease the chemotherapy side effects compared to chemotherapy alone [9]. We included and analyzed 16 randomized controlled trials that matched the criteria and found that SFI did improve the antitumor efficacy. Moreover, through mass spectrometry analysis, we found that SFI is rich in flavonoids and terpenoids, which can effectively exert antitumor effects. At the same time, studies have found that compounds such as 7-hydroxycoumarins and Glycosidic flavonoids in SFI components can inhibit the proliferation of A549 cells by interfering with MIF-CD74 interactions [22, 23]. It is precise because of the many effective antitumor components in SFI that help it to play a unique role in tumor treatment, which needs to be further explored by many researchers.

Notably, we confirmed that SFI could significantly attenuate tumor cell viability and inhibit biological functions such as cell proliferation, migration, and invasion, which was coincident with previous research results on SFI [19]. In addition, the four cell lines A549, NCI-H2009, NCI-H226 and SK-MES-1 all had the same 25 genes that were significantly upregulated (|log2-fold change|> 1 and adjusted p < 0.05). Since it includes genes such as CHOP, CTSK and SAT1, SFI was associated with the tumor cell apoptosis pathway and ferroptosis pathway. These dysregulated genes in the pathway might serve as key players in SFI intervention.

We found that CHOP levels were significantly increased after SFI treatment. CHOP is identified as a specific transcription factor in the ERS-mediated apoptosis pathway, which regulates a member of the CCAAT/enhancer-binding protein (C/EBP) family of transcription factors that induce apoptosis [24, 25]. It is well known that Bcl-2 can control mitochondrial apoptosis by regulating mitochondrial outer membrane permeability through multiple domains, such as Bax and Bak [26]. Studies have shown that CHOP can reduce the level of Bcl-2 in NSCLC, thereby inhibiting cell growth and promoting apoptosis [27]. At the same time, reducing the expression of CHOP by siRNA inhibited the expression of PUMA and reduced the level of apoptosis [28]. In our study, compared with the control group, we found that the proapoptotic gene-related proteins Bax and caspase 3 were significantly increased by western blotting assays, while the expression of Bcl-2 was significantly downregulated. Moreover, the negative correlation between CHOP and Bcl-2, and the positive correlation between CHOP and Bax in TCGA database also confirmed that CHOP may be a potential regulator of SFI.

Notably, CHOP has demonstrated clinical predictive value in a variety of tumors. The expression of CHOP has been reported to be related to the prognosis of tumor patients. Zhu et al. showed that low CHOP expression predicts poor prognosis in GCA patients [29]. Studies have found that CHOP, commonly used as a marker of endoplasmic reticulum (ER) stress, has predictive value in breast cancer patients receiving adjuvant chemotherapy, and CHOP is associated with prolonged disease-free survival (HR = 0.385, 95% CI 0.215–0.688; P = 0.001) [30]. In addition, studies have also demonstrated that the level of CHOP is associated with the clinical stage of cancer patients and the status of lymph node metastasis in NSCLC patients [31, 32]. SFI is expected to further improve the prognosis of patients by increasing CHOP levels.

Furthermore, in vivo experiments showed that SFI significantly inhibited the growth of NSCLC xenograft tumors. Taken together, these data demonstrated that SFI reduced cell viability, inhibited cell migration and invasion, regulated the cell cycle and apoptosis levels, and controlled the in vitro and in vivo growth state of NSCLC cells by enhancing CHOP-mediated apoptosis activation. A total of 3988 genes that were upregulated after SFI stimulation were identified by mRNA-seq in A549, NCI-H2009, NCI-H226 and SK-MES-1 cells, of which 25 genes showed consistent upregulated changes in different cell lines. Furthermore, based on KEGG enrichment, dysregulated SFI-related genes in NSCLC cells were significantly associated with some pathways that may play important roles (e.g., apoptosis, ferroptosis, transcriptional dysregulation in immune-related pathways, and multiple signaling pathways). These data could deepen our understanding of NSCLC treatment with SFI from the perspective of molecular mechanisms. Additional exploration of key targets and pathways (e.g., transcriptional dysregulation in cancer, rheumatoid arthritis, and the cGMP-PKG signaling pathway) may help to better exploit the role of SFI in future cancer treatments.

## Conclusion

In summary, SFI could markedly improve the chemotherapy efficacy of NSCLC patients. The main active ingredients include flavonoids and terpenoids, which can effectively exert antitumor effects. SFI hindered tumor progression and might promote apoptosis by increasing the expression of Bax, caspase 3 and decreasing the level of Bcl-2 in NSCLC.

### Supplementary Information

Supplementary material 1.

## Data Availability

The research data generated from this study are included in the article.

## Abbreviations

SFI: Shenqi Fuzheng Injection
NSCLC: Non-small cell lung cancer
UPLC‒MS: Ultra performance liquid chromatography-mass spectrometer
CCK-8: Cell Counting Kit-8
LC: Lung cancer
LUAD: Lung adenocarcinoma
LUSC: Lung squamous carcinoma
ORR: Objective response rate
IDA: Information-dependent acquisition
DP: Declustering otential
S.D.: Standard deviation
ESI: Essential science indicators
KEGG: Kyoto Encyclopedia of Genes and Genomes
TCGA: The Cancer Genome Atlas
mRNA-seq: Message RNA-sequencing
C/EBP: CCAAT/enhancer-binding protein
ER: Endoplasmic reticulum
