# Supplementary material for: Shenqi Fuzheng injection hinders non-small cell lung cancer cell growth by regulating the Bax/Bcl-2 signaling pathway
Source: Discov Oncol. 2024 May 29;15:195. doi: 10.1007/s12672-024-01029-6 (PMC11136924; doi:10.1007/s12672-024-01029-6)
Supplement: Supplementary file 1 — Supplementary material 1. [file 12672_2024_1029_MOESM1_ESM.docx]

**Supplementary materials**


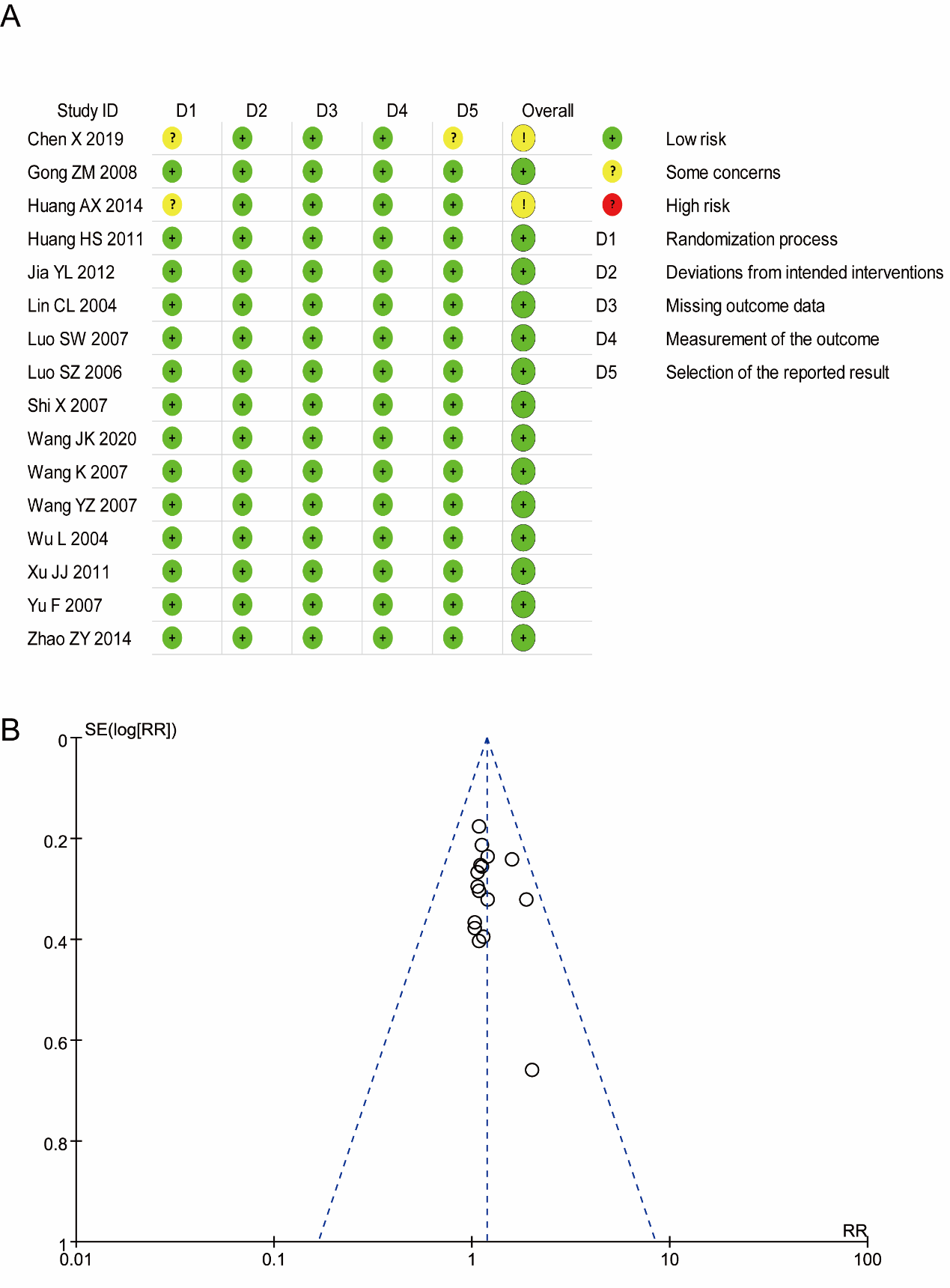


Supplementary Fig. 1. (A) The assessment of risk-of-bias for randomized trials. (B) The funnel plot is based on studies with data on objective tumor response.


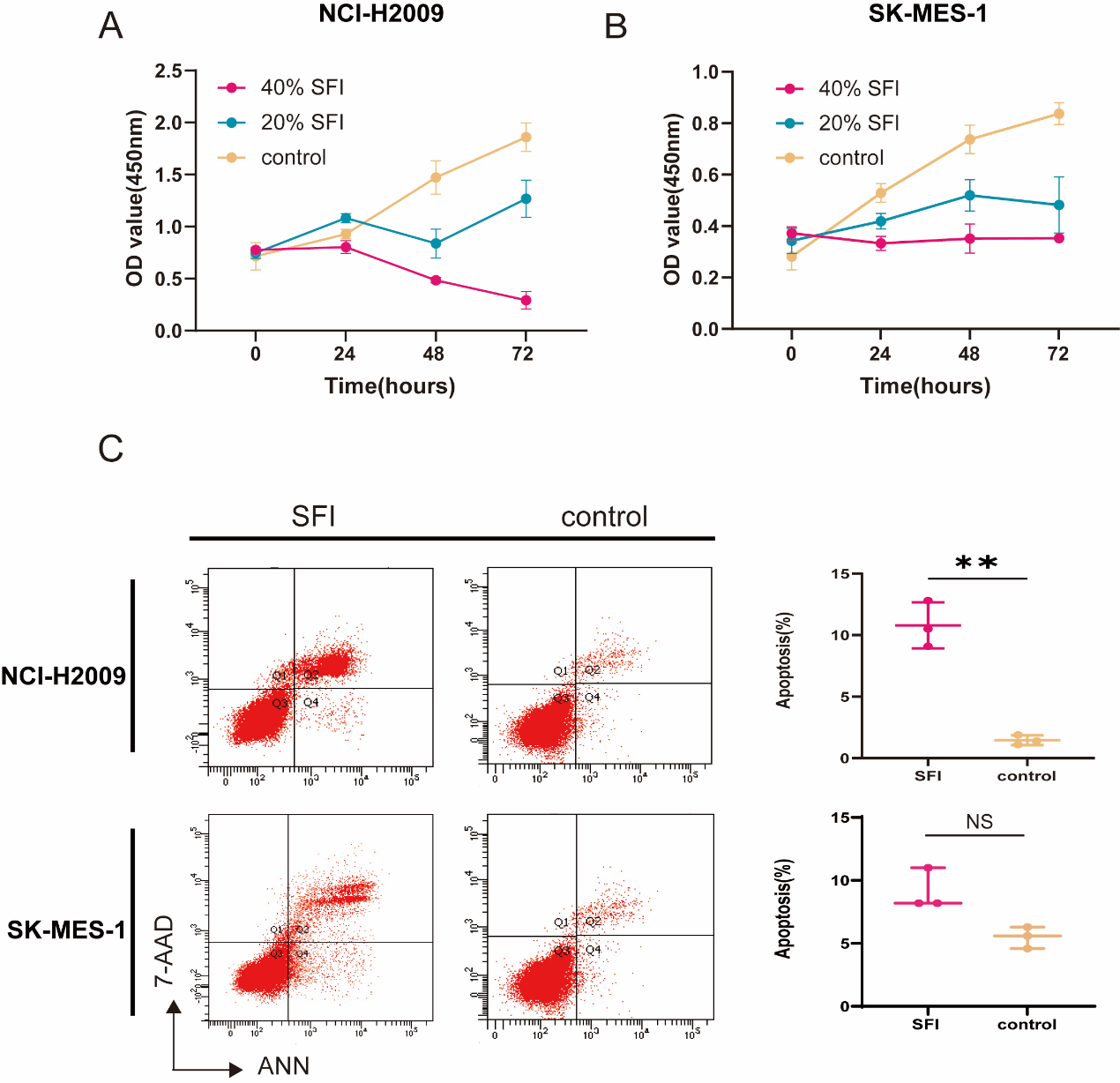


Supplementary Fig. 2. NCI-H2009 (A) and SK-MES-1 (B) cells viability were measured by cell counting kit 8 assay after treatment with Shenqi Fuzheng Injection at different concentrations for 24, 48, and 72 h. (C) The apoptosis of SFI-treated NCI-H2009 and SK-MES-1 cells was analyzed by flow cytometry. *p < 0.05, **p < 0.01, ***p < 0.001. (SFI, Shenqi Fuzheng Injection.)


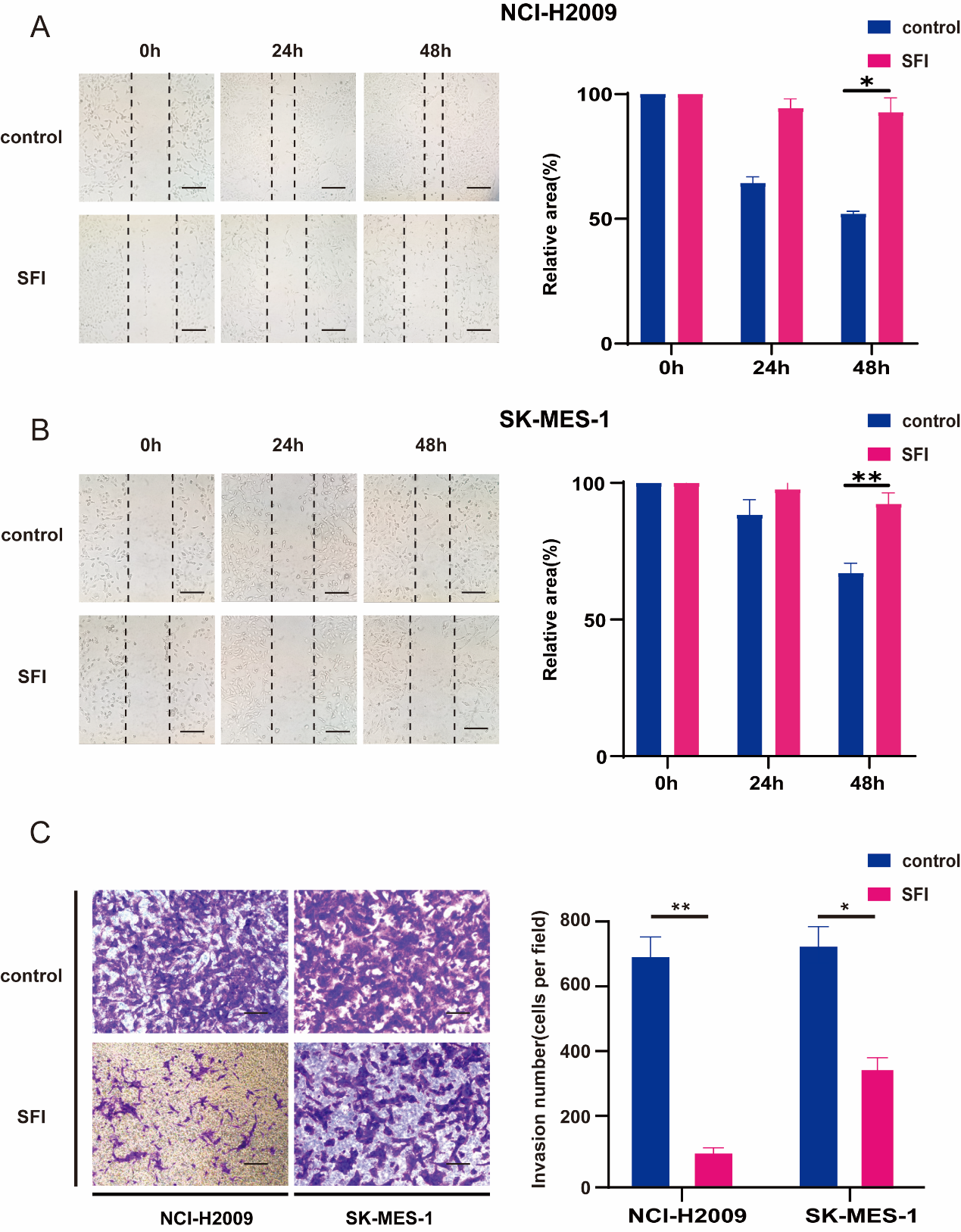
Supplementary Fig. 3. NCI-H2009 (A) and SK-MES-1 (B) cells were treated with SFI for the migration assay. (C) NCI-H2009 and SK-MES-1 cells mobility were measured using the Transwell system as described in Materials and Methods. *p < 0.05, **p < 0.01, ***p < 0.001. (SFI, Shenqi Fuzheng Injection.)

**Supplementary Table 1 Randomized controlled trials about SFI included in the Meta-analysis**

| **Number** | **Studies** | **Studies**  **(Author)** | **Year** | **Number**  **(T/C)** | **KPS** | **Stage** | **Chemotherapy regimen** | | **Endpoint** | **ORR** | |
| --- | --- | --- | --- | --- | --- | --- | --- | --- | --- | --- | --- |
|  |  |  |  |  |  |  | **T** | **C** |  | **T** | **C** |
| 1 | Clinical observation of senile non-small cell lung cancer treated with adjuvant chemotherapy by Shenqi Fuzheng injection | Chenglin Liu | 2004 | 60/60 | ≥60 | Ⅱ-Ⅳ | SFI+NP(SFI 250mL/d) | NP | Tumor response | 45.00% | 40.00% |
| 2 | Shenqi Fuzheng injection combined with chemotherapy in the treatment of 30 patients with advanced non-small cell lung cancer | Li Wu | 2004 | 30/30 | ≥50 | Ⅲ-Ⅳ | SFI+TC/TP(SFI 250mL/d) | TC/TP | Tumor response | 43.30% | 40.00% |
| 3 | Clinical observation of Shenqi Fuzheng injection combined with paclitaxel and cisplatin in the treatment of advanced non-small cell lung cancer | Shizheng Luo | 2006 | 25/25 | ≥60 | Ⅲ-Ⅳ | SFI+TP(SFI 250mL/d) | TP | Tumor response | 48.00% | 40.00% |
| 4 | Clinical observation of Shenqi Fuzheng injection combined with paclitaxel plus cisplatin in treatment of advanced non-small cell lung cancer | Shewen Luo | 2007 | 30/30 | ≥60 | Ⅲ-Ⅳ | SFI+TP(SFI 250mL/d) | TP | Tumor response | 60.00% | 50.00% |
| 5 | Shenqi Fuzheng injection combined with chemotherapy for advanced non-small cell lung cancer | Xing Shi | 2007 | 32/27 | ≥60 | Ⅲ-Ⅳ | SFI+NP(SFI 250mL/d) | NP | Tumor response | 34.40% | 33.30% |
| 6 | Clinical observation of Shenqi Fuzheng injection combined with NP therapy in the treatment of late stage non-small cell lung cancer | Kun Wang | 2007 | 18/18 | ≥50 | Ⅲ-Ⅳ | SFI+NP(SFI 250mL/d) | NP | Tumor response | 50.00% | 39.00% |
| 7 | Clinical observation of Shenqi Fuzheng injection combined with vinorelbine and carboplatin in the treatment of advanced non-small cell lung cancer | Yuzhou Wang | 2007 | 28/28 | ≥60 | Ⅲ-Ⅳ | SFI+NP(SFI 250mL/d) | NP | Tumor response | 32.14% | 29.63% |
| 8 | Clinical observation of Shenqi Fuzheng injection adjuvant chemotherapy in the treatment of non-small cell lung cancer | Fei Yu | 2007 | 30/30 | ≥50 | Ⅲ-Ⅳ | SFI+DC(SFI 250mL/d) | DC | Tumor response | 50.00% | 46.70% |
| 9 | Curative effect of Shenqi Fuzheng injection combined with NP chemotherapy in elderly patients with advanced non-small cell lung cancer | Zhimin Gong | 2008 | 33/32 | ≥60 | Ⅲ-Ⅳ | SFI+NP(SFI 250mL/d) | NP | Tumor response | 51.50% | 46.90% |
| 10 | Clinical observation of Shenqi Fuzheng injection combined with gemcitabine and cisplatin in the treatment of advanced non-small cell lung cancer in elderly patients | Jinjun Xu | 2011 | 30/30 | ≥70 | Ⅲ-Ⅳ | SFI+GP(SFI 250mL/d) | GP | Tumor response | 38.50% | 35.90% |
| 11 | Efficacy of Chinese medicine combined with gemcitabine chemotherapy in the treatment of advanced non-small cell lung cancer and its effect on serum MMP 9 | Hansheng Huang | 2011 | 39/39 | NA | Ⅲ-Ⅳ | SFI+GP(SFI 250mL/d) | GP | Tumor response | 56.70% | 30.00% |
| 12 | Enhancement of Shenqi Fuzheng injection solution in elderly patients with late stage non-small cell lung cancer treated with chemotherapy | Yanling Jia | 2012 | 72/71 | ≥60 | Ⅲ-Ⅳ | SFI+NP(SFI 250mL/d) | NP | Tumor response | 48.60% | 45.10% |
| 13 | Efficacy of Shenqi Fuzheng combined with PG regimen in the treatment of advanced non-small cell lung cancer | Aixia Huang | 2014 | 38/38 | ≥70 | Ⅲ-Ⅳ | SFI+GP(SFI 250mL/d) | GP | Tumor response | 47.37% | 42.11% |
| 14 | Clinical observation of Shenqi Fuzheng injection improving the adverse reactions of chemotherapy in advanced non-small cell lung cancer | Zhenyu Zhao | 2014 | 50/52 | ≥60 | Ⅲ-Ⅳ | SFI+GP(SFI 250mL/d) | GP | Tumor response | 22.00% | 21.00% |
| 15 | Preventive effect of Shenqi Fuzheng injection on adverse reactions in adjuvant treatment of advanced non-small cell lung cancer patients undergoing chemotherapy | Xuan chen | 2019 | 30/30 | NA | NA | SFI+GP(SFI 250mL/d) | GP | Tumor response | 20.00% | 10.00% |
| 16 | Clinical study of Shenqi Fuzheng injection combined with chemotherapy in the treatment of non-small cell lung cancer | Jingke Wang | 2020 | 40/40 | NA | Ⅱ-Ⅳ | SFI+GP(SFI 250mL/d) | GP | Tumor response | 60.00% | 37.50% |

**Supplementary Table 2 UPLC-MS/MS to identify the effective ingredients of Senqi Fuzheng Zhusheye**

| No. | RT (min) | Adduct | Precursor m/z | Molecular formula | Ontology |
| --- | --- | --- | --- | --- | --- |
| 1 | 1.37672 | [M+H]+ | 365.0802 | C_18_H_17_ClO_6_ | Hydroxybenzoic acid derivatives |
| 2 | 1.37672 | [M+H]+ | 203.0383 | C_11_H_6_O_4_ | 5-hydroxypsoralens |
| 3 | 1.37672 | [M+Na]+ | 185.0284 | C_9_H_6_O_3_ | 7-hydroxycoumarins |
| 4 | 4.68897 | [M+H]+ | 549.155 | C_26_H_28_O_13_ | Isoflavonoid C-glycosides |
| 5 | 5.0397 | [M+H]+ | 611.2083 | C_28_H_34_O_15_ | Flavonoid-7-O-glycosides |
| 6 | 5.56515 | [M+H]+ | 285.0563 | C_16_H_10_N_2_O_2_ | Indolines |
| 7 | 5.61787 | [M-H]- | 491.1565 | C_23_H_24_O_12_ | Flavonoid O-glycosides |
| 8 | 5.64482 | [M+H]+ | 449.1681 | C_19_H_28_O_12_ | Iridoid O-glycosides |
| 9 | 5.77635 | [M-H]- | 425.2379 | C_23_H_22_O_8_ | Rotenones |
| 10 | 5.95097 | [M+Na]+ | 419.157 | C_22_H_24_N_2_O_5_ | Psoralens |
| 11 | 6.70085 | [M+Na]+ | 617.1433 | C_27_H_30_O_15_ | Flavonoid O-glycosides |
| 12 | 6.91273 | [M+Na]+ | 485.1097 | C_22_H_22_O_11_ | Flavonoid-7-O-glycosides |
| 13 | 6.9408 | [M-H]- | 507.1881 | C_25_H_32_O_11_ | Iridoid O-glycosides |
| 14 | 7.85465 | [M+FA-H]- | 991.5667 | C_48_H_82_O_18_ | Triterpene saponins |
| 15 | 8.13557 | [M+FA-H]- | 829.5112 | C_42_H_72_O_13_ | Triterpene saponins |
| 16 | 8.47483 | [M+H]+ | 849.4034 | C_42_H_66_O_15_ | Triterpene saponins |
| 17 | 8.77865 | [M+H]+ | 849.4044 | C_42_H_66_O_16_ | Triterpenoids |
| 18 | 9.0012 | [M-H]- | 871.5236 | C_46_H_81_O_13_P | Phosphatidylinositols |
| 19 | 9.06728 | [M+H]+ | 274.2539 | C_19_H_31_N | Phenylpropanes |
| 20 | 9.51578 | [M+Na]+ | 891.4099 | C_49_H_60_N_2_O_12_ | Macrolactams |
| 21 | 9.6361 | [M+H]+ | 437.1638 | C_19_H_18_N_4_O_7_ | 5'-deoxyribonucleosides |
| 22 | 13.5286 | [M-H]- | 333.2625 | C_20_H_30_O_4_ | Diterpenoids |
| 23 | 14.1564 | [M+H]+ | 184.0597 | C_8_H_9_NO_4_ | Pyridinecarboxylic acids |
